# Slit diaphragm maintenance requires dynamic clathrin-mediated endocytosis facilitated by AP-2, Lap, Aux and Hsc70-4 in nephrocytes

**DOI:** 10.1186/s13578-021-00595-4

**Published:** 2021-05-11

**Authors:** Luyao Wang, Pei Wen, Joyce van de Leemput, Zhanzheng Zhao, Zhe Han

**Affiliations:** 1 Department of Nephrology, The First Affiliated Hospital of Zhengzhou University, No. 1 Jianshe Eastern Road, Zhengzhou, 450052 Henan China; 2 Center for Precision Disease Modeling, Department of Medicine, University of Maryland School of Medicine, 670 West Baltimore Street, Baltimore, MD 21201 USA; 3 Division of Endocrinology, Diabetes and Nutrition, Department of Medicine, University of Maryland School of Medicine, Baltimore, MD 21201 USA

**Keywords:** *Drosophila*, Nephrocyte, Slit diaphragm, Endocytosis, Clathrin, Shi, AP-2 complex, Lap, Auxilin, Hsc70-4

## Abstract

**Background:**

The Slit diaphragm (SD) is the key filtration structure in human glomerular kidney that is affected in many types of renal diseases. SD proteins are known to undergo endocytosis and recycling to maintain the integrity of the filtration structure. However, the key components of this pathway remain unclear.

**Methods:**

Using the *Drosophila* nephrocyte as a genetic screen platform, we screened most genes involved in endocytosis and cell trafficking, and identified the key components of the cell trafficking pathway required for SD protein endocytosis and recycling.

**Results:**

We discovered that the SD protein endocytosis and recycling pathway contains clathrin, dynamin, AP-2 complex, like-AP180 (Lap), auxilin and Hsc70-4 (the endocytosis part) followed by Rab11 and the exocyst complex (the recycling part). Disrupting any component in this pathway led to disrupted SD on the cell surface and intracellular accumulation of mislocalized SD proteins. We also showed the first in vivo evidence of trapped SD proteins in clathrin-coated pits at the plasma membrane when this pathway is disrupted.

**Conclusions:**

All genes in this SD protein endocytosis and recycling pathway, as well as SD proteins themselves, are highly conserved from flies to humans. Thus, our results suggest that the SD proteins in human kidney undergo the same endocytosis and recycling pathway to maintain the filtration structure, and mutations in any genes in this pathway could lead to abnormal SD and renal diseases.

**Supplementary Information:**

The online version contains supplementary material available at 10.1186/s13578-021-00595-4.

## Introduction

The glomerular filtration barrier, which contains the fenestrated endothelium of the glomerular capillaries, the glomerular basement membranes and podocytes, prevents passage of the majority of proteins, such as albumin, from blood into urine. Damages to the glomerulus causes proteinuria, wherein proteins in the blood leak into the urine, and may progress into kidney failure. The mammalian podocyte slit diaphragm structure is a major component of the glomerular filtration barrier, indeed its disruption has been associated with proteinuria and many types of renal diseases [21]

Nephrin, a transmembrane protein encoded by the *NPHS1* gene [15], plays an essential role in assembly of the slit diaphragm structure and also functions as a signaling platform regulating the podocyte actin cytoskeleton, membrane trafficking and calcium mechano-signaling [17]. Mutations in *NPHS1* disrupt slit diaphragm structure, cause foot process effacement and lead to severe proteinuria and nephrotic syndrome [15, 26]. Perturbations in nephrin protein level and localization have been found in podocytes of patients with membranous glomerulonephritis (GN), minimal change GN and diabetic nephropathy [4, 5], suggesting regulation of nephrin is critical for slit diaphragm function. The cytoplasmic tail of nephrin, when phosphorylated by Src-family tyrosine kinases, can selectively bind Nck adaptor proteins which regulate the cytoskeleton, thus linking nephrin to the podocyte actin cytoskeleton [13, 30].

In cultured cells, nephrin, when dephosphorylated at the conserved Y1193 residue, was shown to interact with β-Arrestin2 and to undergo endocytosis to attenuate its signaling [23]. Similarly in COS-7 cells, nephrin was found to be endocytosed through both clathrin-mediated endocytosis (CME) and raft-mediated endocytosis (RME), which is clathrin-independent yet like CME requires dynamin activity [22]. Using different pharmacological inhibitors, Waters et al. found that Notch promotes dynamin-dependent, raft-independent endocytosis of nephrin [33]. The above studies were carried out in cultured cells, therefore while the results have provided possible mechanisms in the regulation of nephrin endocytosis, it remained unclear whether nephrin and other slit diaphragm proteins are regulated similarly in podocytes in vivo.

It has been shown in mice, that podocyte-specific ablation of dynamin, synaptojanin and endophilin—all three critical components of clathrin-mediated endocytosis—results in endocytic defects, foot process effacement, severe proteinuria and kidney failure [25]. Recently, disease-associated variants in other endocytosis/recycling pathway genes, like GAPVD1, ANKFY1, TBC1D8B and EXOC4, have been identified in patients with nephrotic syndrome, suggesting that endocytosis and recycling play an important role in glomerular function [3, 12, 14]. However, it is challenging to systematically examine the localization of nephrin proteins in mammalian kidney samples with endocytic/recycling mutations at high resolution [27, 28], thereby limiting the scope of the mammalian system. As such it remains unclear whether the endocytosis/recycling pathway directly regulates the dynamics of slit diaphragm proteins in vivo.

The *Drosophila* nephrocyte, fly equivalent of podocyte, provides an excellent model system to study the dynamics of slit diaphragm proteins in vivo. We and others have found that over 85% of the genes involved in human steroid resistant nephrotic syndrome have functional homologs in *Drosophila* nephrocytes [6, 11]. The *Drosophila* nephrocyte slit diaphragm (NSD) has been shown to share functional, molecular and ultrastructural similarities to the mammalian podocyte slit diaphragm [34, 37]. The distribution of *Drosophila* NSD proteins at nephrocyte plasma membrane is highly organized in a fingerprint-like pattern and is sensitive to genetic perturbations as in mammals [11, 34], providing an excellent assay to probe the conserved mechanisms to regulate the dynamics of slit diaphragm proteins. Moreover, we recently established an endocytosis and recycling model for slit diaphragm proteins in *Drosophila* [35]. We showed that silencing the critical players in the endocytosis and recycling pathways led to severe defects in nephrocyte function, disruption of slit diaphragm protein localization and loss of the NSD ultrastructure. For example, the exocyst proteins, which form a conserved complex tethering exocytosis vesicles or recycling endosomes to the plasma membrane, are required for slit diaphragm protein localization in nephrocytes [35], which is consistent with the results that mutations of human *EXOC4* and mouse *EXOC5* lead to severely impaired kidney filtration [19]. Silencing *Rab5* and *Rab11*, which are required for the early and late endosome functions, respectively, led to slit diaphragm protein mislocalization. We proposed a working model in which slit diaphragm proteins are endocytosed from NSD structures at the nephrocyte plasma membrane, then enter the Rab5-dependent early endosome and are subsequently sorted into Rab11-dependent recycling endosomes. Aided by the exocyst complex, the recycling endosome was tethered to the surface membrane, and via membrane fusion returned the slit diaphragm proteins once again to the NSD structures.

While CME is the dominant and well-studied endocytic route, CIE routes, which include caveolar pathway, clathrin-independent carriers/GPI-AP enriched early endosomal compartments (CLIC/GEEC) pathway and other pathways, are emerging as important regulators of cell growth and development. Since vesicles from both CME and CIE pathways can enter the early endosomes, it was not clear which pathway, CME or CIE, is required for slit diaphragm protein endocytosis and NSD structure integrity. Here, we screened candidate genes involved in both CME and CIE in the nephrocytes and found that the CME pathway is essential for maintenance of the NSD structure.

## Results

### Clathrin is required for the endocytosis of slit diaphragm proteins in nephrocytes

**Fig. 1:**
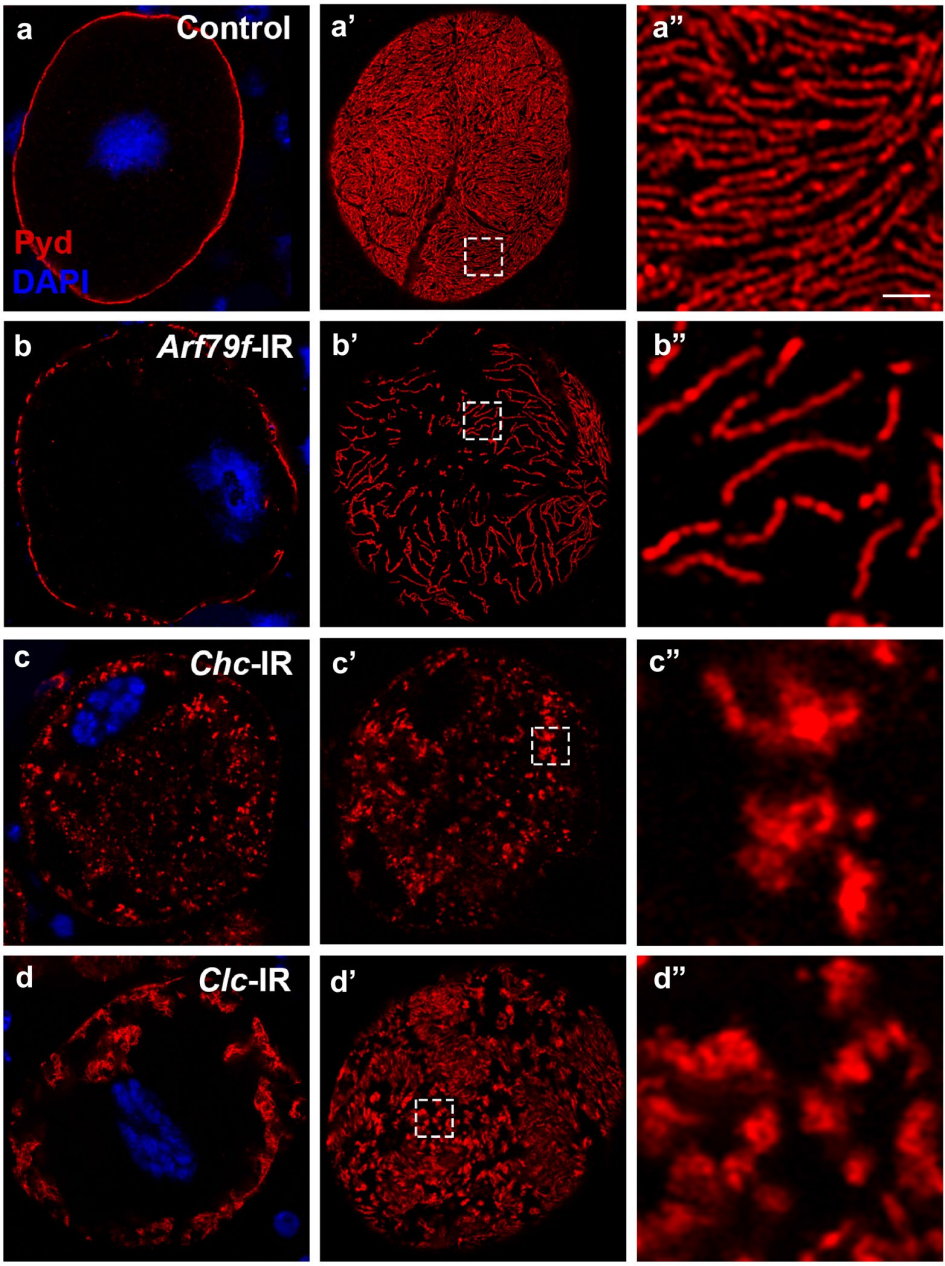
The clathrin complex is required for slit diaphragm protein endocytosis. Immunofluorescence labeling for Pyd (red), with DAPI (blue) nuclear stain of adult fly nephrocytes, visualized by confocal microscopy. Scale bar: 1 μm. **a–a″** Wild type (Control) adult fly nephrocytes exhibited Pyd (red) localization by immunofluorescence labeling that was tightly localized to the cell margin, defining a highly regular, continuous circumferential ring when examined by confocal microscopy (**a**, medial optical section). Visualization of Pyd at the cell surface plane showed a highly regular and uniform pattern of distribution (**a′**–**a″**). Cell nuclei were labeled by DAPI (blue). Scale bar: 1 μm. **b** Silencing of Arf79f (**b**–**b**″) did not cause intracellular accumulation of Pyd proteins. But on the cell surface section, the distance between the lines of Pyd fingerprint become wider. **c**–**d** Silencing of *Chc* (**c**–**c″**) or *Clc* (**d**–**d″**) genes of the clathrin complex resulted in a severe disruption of normal Pyd localization at the nephrocyte cell margin and the cell surface. Intracellular Pyd showed a distribution with aggregate-like concentrates. The fingerprint pattern of Pyd at the cell surface was also disrupted: the highly organized and evenly spaced lines were gone, replaced by irregular short lines or aggregates

Our previous work showed that silencing genes required for slit diaphragm proteins endocytosis and recycling disrupts NSD structures and leads to mislocalization of slit diaphragm proteins (like Sns and Pyd, the fly homologs of Nephrin and ZO-1, respectively). Utilizing this specific phenotype, we systematically screened candidate endocytic genes for their roles in the NSD structure maintenance (Additional file 1: Table S1). We used the Dot-Gal4 nephrocyte-specific driver to express the RNA knock down transgenes, UAS-RNAi. The knockdown was usually efficient and specific, as shown by antibody staining of several proteins (Additional file 1: Figure S4 and Figure S10). The clathrin-independent endocytosis (CIE) is relatively understudied in *Drosophila*. For example, there is no fly homolog for Caveolin, so it is unclear whether fly has caveolar endocytosis pathway. And many CIE routes identified in mammalian cells have not been studied in *Drosophila* cells [9]. However, the CLIC/GEEC pathway, which is the route for uptake of glycosylphosphatidylinositol-anchored proteins (GPI-APs) and fluid phase, is conserved and the only CIE route that has been molecularly characterized and confirmed in *Drosophila* [7, 9, 10]. We tested the phenotype of RNAi knockdown of the critical factor in the CLIC/GEEC pathway, Arf79F, fly homolog of mammalian ARF1, in nephrocytes [7, 16]. In wild type nephrocytes imaged in medial plane section across the nucleus, immune-labeling of Pyd revealed its strong association with cell membrane, with few signal inside the cell (Fig. 1a; on the cell surface section, Pyd showed a uniform and smoothly distributed fingerprint-like localization pattern (Fig. 1a′–a′′). Silencing Arf79F did not affect the lines of the fingerprint pattern of Pyd localization, but the gap between the lines increased significantly, causing reduced total surface density of Pyd (Fig. 1b′–b′′). However, Pyd protein did not accumulate inside the nephrocytes (Fig. 1b). This phenotype is different from known genes involved in NSD endocytosis and recycling, such as exocyst [35]. As Arf79F is also involved in other cellular processes such as Golgi trafficking, it is unclear whether this Pyd phenotype was caused by defects in the CLIC/GEEC pathway.

To directly test the role of CME in slit diaphragm protein endocytosis, we silenced *Clathrin heavy chain* (*Chc*) and *Clathrin light chain* (*Clc*) in nephrocytes. The lack of Chc or Clc resulted in mislocalized slit diaphragm protein Pyd. Inside the nephrocytes, aggregates were accumulated; on the cell surface, the characteristic Pyd fingerprint-like pattern was disrupted and some aggregates were formed (Fig. 1c–d′′). The localization of another slit diaphragm protein Sns was similarly disrupted (Additional file 1: Figure S2).The phenotype of clathrin deficient nephrocytes is highly similar to that of nephrocytes deficient with exocyst genes [35], suggesting they function in the same pathway for slit diaphragm protein endocytosis and recycling. The above results demonstrate that clathrin is directly required for slit diaphragm protein endocytosis.

**Fig. 2:**
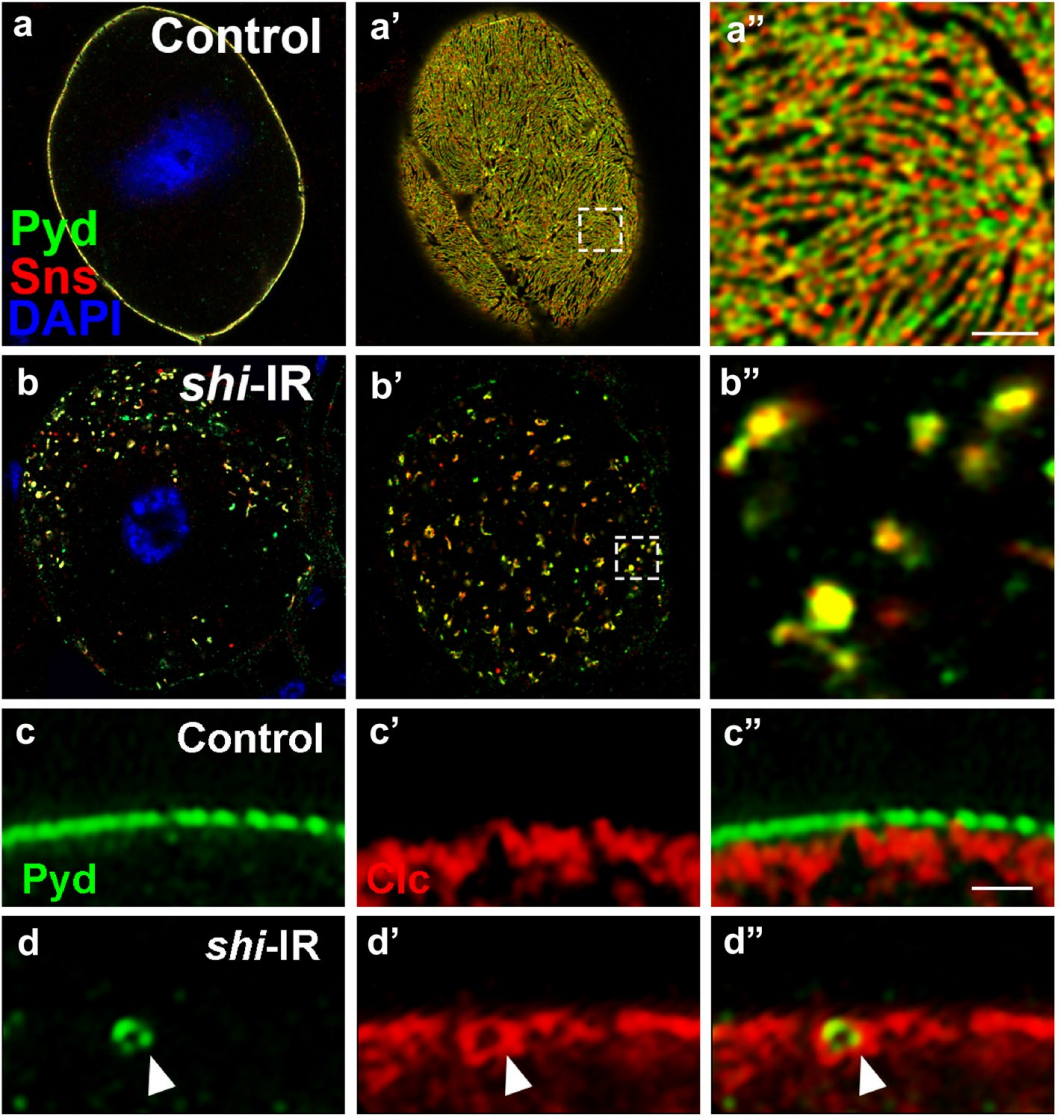
The endocytosis regulator, Shi, is required for nephrocyte function and localization of slit diaphragm protein. **a** Immunofluorescent labeling in wild type (Control) adult fly nephrocytes exhibited tight co-localization of Pyd (green) and Sns (red) at the cell margin, defining a fine and sharply continuous ring when examined by confocal microscopy (**a**, medial optical section). Detection of Pyd and Sns (**a′**–**a″**) at the cell surface plane showed a highly regular and fingerprint-like distribution pattern. Cell nuclei are labeled by DAPI (blue). Scale bar: 1 μm. **b**–**b″** Immunofluorescent labeling in *shi*-silenced (*shi*-IR) adult fly nephrocytes revealed disturbed Pyd and Sns localization at the nephrocyte cell margin and were found to have accumulated intracellularly with appearance of aggregates (**b**). The characteristic cell surface localization had been severely disrupted, visible as dots of seemingly Pyd and Sns aggregates and overall cell surface expression of Pyd and Sns was greatly reduced (**b′**–**b″**). **c**–**d** Immunofluorescence labeling of Pyd (green) and Clc (red). In wild type (Control) nephrocytes (**c**–**c″**), Pyd localized at the cell membrane. Clc localized to vesicles adjacent to the cell membrane. In *shi*-silenced (*shi*-IR) nephrocytes (**d**–**d″**), Pyd was mostly absent at the cell membrane, but can be occationally found in extraordinary large Clathrin-coated vesicles (arrowhead). Scale bar: 1 μm

We tested whether clathrin is required for nephrocyte endocytosis and reabsorption function by in vivo protein uptake assay. In flies carrying *MHC-ANF-RFP*, the myosin heavy chain (MHC) promoter directs muscle cell expression of a rat atrium natriuretic factor (ANF)–red fluorescent protein (RFP) fusion protein (ANF-RFP) that is secreted into the hemolymph. ANF-RFP is typically taken up by healthy, wild type nephrocytes and the intracellular red fluorescence can be readily visualized and quantitated in vivo (Additional file 1: Figure S1A). Silencing of *chc* or *clc* led to severely reduced levels of RFP fluorescence within nephrocytes (Additional file 1: Figure S1A, C), indicating that clathrin is required for the hemolymph protein filtering/absorption functions of nephrocyte. In an independent ex vivo functional assay, we tested the ability of dissected nephrocytes to absorb Texas Red-labeled 10 kD Dextran particles. Again, silencing of *chc* or *clc* induced a marked deficiency in intracellular Texas Red fluorescence compared to control wildtype nephrocytes (Additional file 1: Figure S1B, D). These observations indicate that clathrin is required for normal nephrocyte function.

### Drosophila shibire, the fly homolog of dynamin, is required for slit diaphragm protein endocytosis

Dynamin, a critical component of CME and some routes of CIE, has been shown to be essential for podocyte function in mice [25]. Its fly homolog Shibire (Shi) is highly conserved and plays important roles in vesicular transport, such as endocytosis, in *Drosophila* [1, 29, 31, 32]. In our screen, we found Shi is also required for slit diaphragm protein endocytosis. Silencing *shi* led to disruption of Sns/Pyd localization: Sns/Pyd aggregated in puncta both inside nephrocytes (Fig. 2b) and on the cell surface (Fig. 2b′, b″), with less cell surface area was covered with Sns/Pyd, similar phenotypes with silencing other genes required for slit diaphragm protein endocytosis and recycling. The results indicate that Shi is required for the maintenance of slit diaphragm structure, through the endocytosis of slit diaphragm proteins.

We tested whether Shi is required for nephrocyte function by nephrocyte-specific silencing of *shi*, followed by in vivo protein uptake assay to examine effects on nephrocyte functioning. Silencing of *shi* led to severely reduced levels of RFP fluorescence within nephrocytes (Additional file 1: Figure S3A, B), indicating that similar to dynamin, Shi is required for the hemolymph protein filtering/absorption functions of nephrocyte. In an independent ex vivo functional assay, we tested the ability of dissected nephrocytes to absorb 10 kD Dextran particles. Again, silencing of *shi* induced a marked deficiency in intracellular Texas Red fluorescence compared to control wildtype nephrocytes (Additional file 1: Figure S3C, D). These observations indicate that Shi is required for normal nephrocyte function.

We tried to find direct evidence of slit diaphragm protein undergoing clathrin-mediated endocytosis by examining co-localization between the slit diaphragm protein Pyd and Clathrin (Clc). In the wild type nephrocyte, Pyd localized at the cell membrane while Clc appeared localized in vesicles near the cell membrane, with little if any overlapping (Fig. 2c–c″). Endocytosis is a rapid process with only a small portion of slit diaphragm proteins undergoing endocytosis at a given time, making it technically difficult to capture extensive co-localization of Pyd and Clc by imaging. However, we reasoned that by blocking the release of clathrin-coated pits we could catch a snapshot of slit diaphragm proteins undergoing endocytosis. To test this hypothesis, we silenced *shi*, which is required for fission of clathrin-coated pits, and then examined the localization of Pyd and Clc. Indeed, we were able to capture clear images of the Pyd protein on a small ring circled by a bigger ring with the Clathrin protein on the cytoplasmic side of the nephrocyte membrane (Fig. 2d–d″), providing the first in vivo evidence that slit diaphragm proteins undergo clathrin-mediated endocytosis from the nephrocyte membrane.

### AP-2 complex and Lap, clathrin-coated pit assembly proteins, play a role in endocytosis of slit diaphragm proteins

The cargo proteins undergoing CME are known to be recognized either directly by Adaptor Protein complex 2 (AP-2) or by other adaptor proteins. The AP-2 complex contains four subunits, known as AP-2α, AP-2μ, AP-2σ and AP-1-2β in *Drosophila*. AP-1-2β is shared between the AP-2 and AP-1 complexes. In fly, the AP-2 complex is involved in plasma membrane endocytosis, while the AP-1 complex is specific for trafficking between the trans-Golgi network and endosomes. Furthermore, the AP-2 complex has been shown to bind plasma membrane phospholipid headgroups and the clathrin complex, and to play a critical role in the assembly of the clathrin-coated pits [20].

**Fig. 3:**
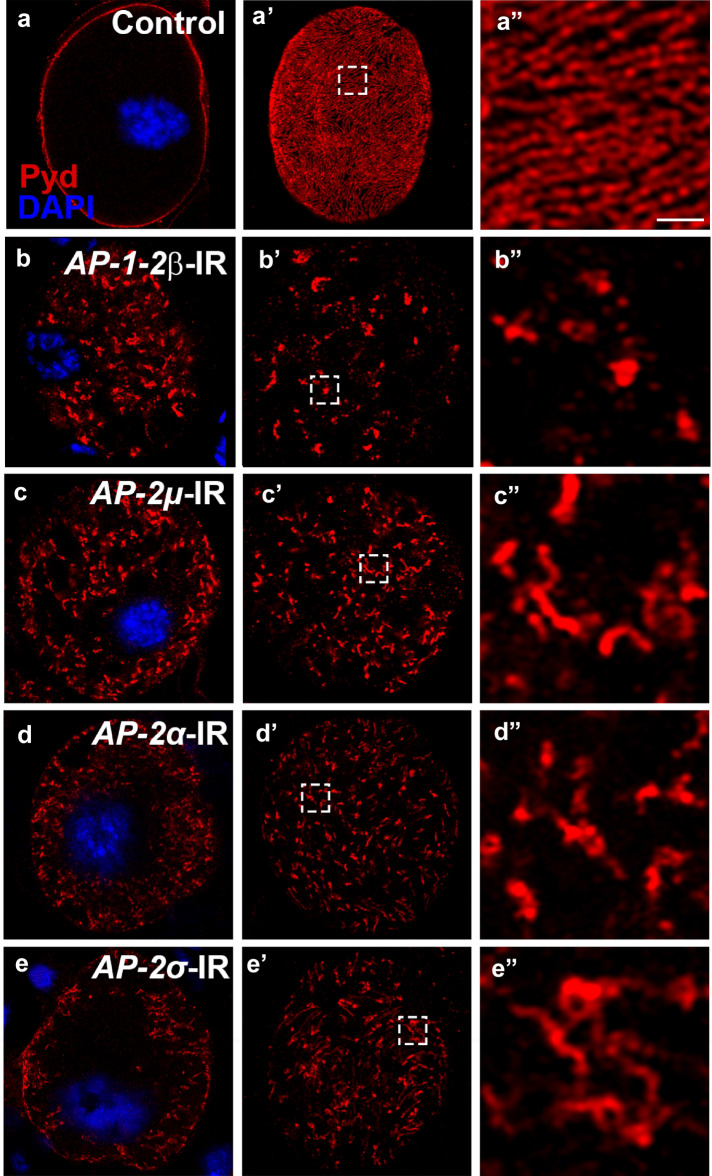
The AP-2 complex, a regulator of clathrin, is required for endocytosis of slit diaphragm protein Pyd. Immunofluorescence labeling for Pyd (red), with DAPI (blue) nuclear stain of adult fly nephrocytes, visualized by confocal microscopy. Scale bar: 1 μm. **a**–**a″** In wild type (Control) nephrocytes, Pyd was localized to the cell membrane (**a**) and exhibited a uniform and smoothly distributed fingerprint-like pattern on the cell surface (**a′**–**a″**). **b**–**e** Silencing of *AP-1-2β* (**b**–**b″**), *AP-2μ* (**c**–**c″**), *AP-2α* (**d**–**d″**) and *AP-2σ* (**e**–**e″**) resulted in severe disruption of Pyd cell surface localization. Revealing intracellular localization of aggregate-like structures, and cell-surface distribution marked by high-density aggregation areas

Depletion of *AP-2* genes resulted in severely reduced filtering/absorption capacity in the nephrocytes (Additional file 1: Figure S5), as well as disrupted Sns/Pyd localization visible as aggregate formation both at the surface and intracellular (Fig. 3; Additional file 1: Figure S6), a phenotype highly similar to that observed in the *Clathrin* (*Chc* or *Clc*)-silenced nephrocytes. Since AP-1-2β is present in both AP-1 and AP-2 complexes, we could not rule out that both complexes are required for slit diaphragm structure maintenance. To exclude the involvement of AP-1, we also silenced genes specific to the AP-1 complex (*AP-1γ*, *AP-1σ* and *AP-1μ*) and found that the localization of slit diaphragm proteins was unchanged from control wild type nephrocytes (Additional file 1: Figure S7). These findings indicate the AP-2 complex, not AP-1, is required for slit diaphragm endocytosis.

**Fig. 4:**
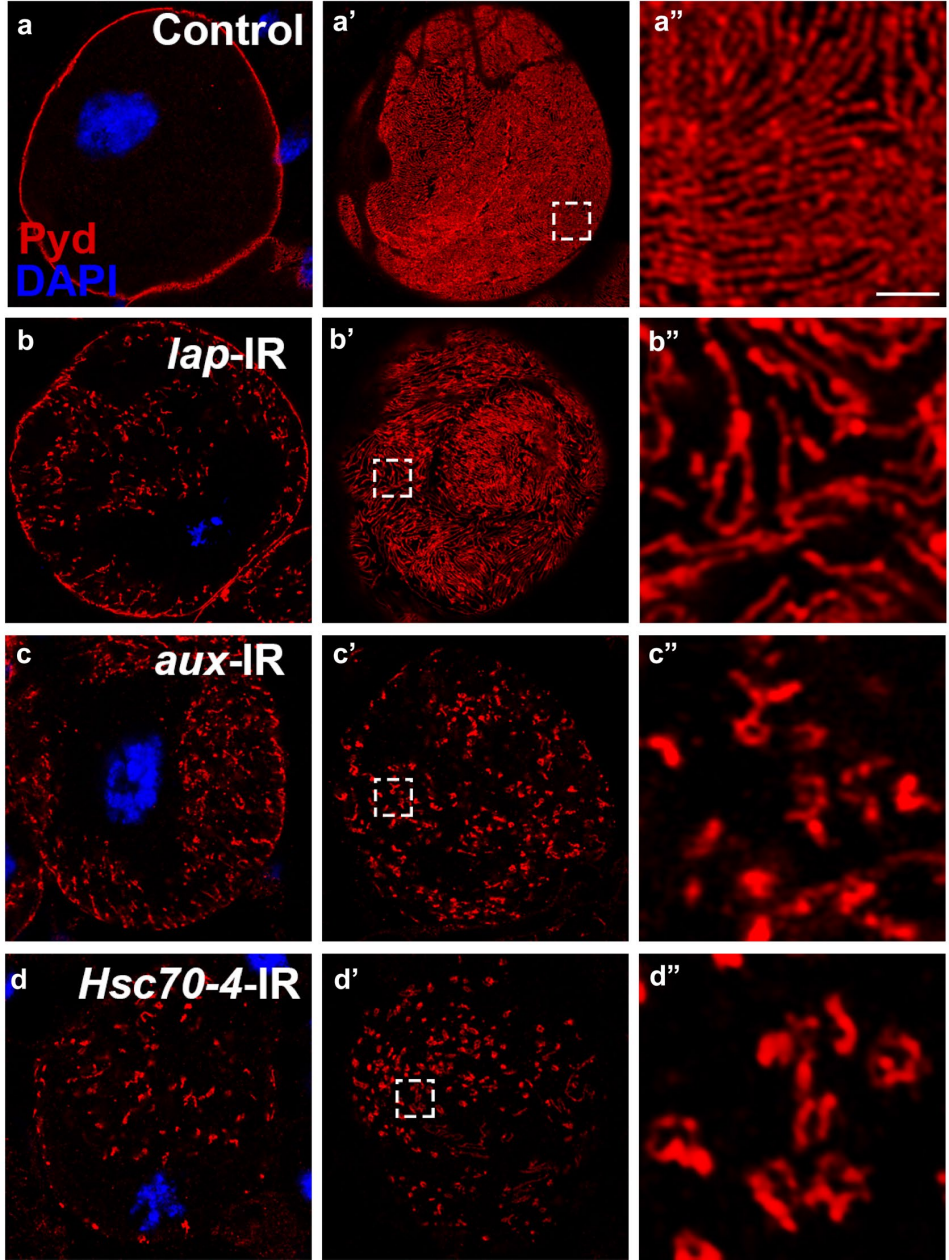
Clathrin accessory proteins for assembly (Lap) and disassembly (Aux; Hsc70-4) are required for endocytosis of slit diaphragm protein Pyd. Immunofluorescence labeling for Pyd (red), with DAPI (blue) nuclear stain of adult fly nephrocytes, visualized by confocal microscopy. Scale bar: 1 μm. **a**–**a**″ In wild type (Control) nephrocytes, Pyd was localized to the cell membrane (**a**) and exhibited a uniform and smoothly distributed fingerprint-like pattern on the cell surface (**a′**–**a″**). **b**–**b″** Silencing of *lap* resulted in intracellular aggregation of Pyd proteins with partial disruption of Pyd cell surface localization. Note the fingerprint-like pattern remained largely intact, however gaps between adjacent Pyd-positive ridges appeared increased. **c**–**d** Silencing of *aux* (**c**–**c″**) and *Hsc70-4* (**d**–**d″**) resulted in severe disruption of Pyd cell surface localization. Revealing intracellular localization of aggregate-like structures, and cell-surface distribution marked by high-density aggregation areas

In addition to the AP-2 complex, other adaptor proteins are required for assembly of the clathrin coat. We examine the role of one such protein, AP180, in slit diaphragm endocytosis. Silencing *like-AP180* (*lap*), the *Drosophila AP180* homolog, in nephrocytes results in a significant reduction in function (Additional file 1: Figure S8). Interestingly, depleted Lap led to accumulation of slit diaphragm protein (Pyd) in the cytoplasm similar to that seen in AP-2 complex gene silencing (Fig. 4b). However, the pattern of Sns/Pyd on the nephrocyte cell surface changed only slightly when *lap* was silenced, with the characteristic fingerprint-like pattern largely intact, although we did observe increased gaps between two slits occasionally (Fig. 4b′–b″, Additional file 1: Figure S9). The results suggest Lap is likely required for the optimal assembly of clathrin coat, but it is not critical for CME pathway activity.

### The Aux and Hsc70-4 clathrin-coated pit disassembly proteins play a role in endocytosis of slit diaphragm proteins

After fission from the plasma membrane, the clathrin-coated vesicles are recognized by auxilin, which then recruits and activates ATPase Hsc70 to remove the clathrin coat. Disassembly of the clathrin coat frees the AP-2 complex, clathrin and Shi (Dynamin) to participate in the next cycle of endocytosis. To examine the role of auxilin and Hsc70 in slit diaphragm protein endocytosis, we silenced their *Drosophila* homologs, *aux* and *Hsc70-4*, in nephrocytes. We found that silencing *Drosophila* homologs, *aux* and *Hsc70-4* in the nephrocytes showed highly similar phenotypes to those of *Clathrin* (*Chc* or *Clc*)-silenced nephrocytes, including a significant reduction in their filtering/absorption function (Additional file 1: Figure S8), and disrupted location of slit diaphragm proteins (Pyd and Sns) (Fig. 4c–d″, Additional file 1: Figure S9). The above results suggest that Aux and Hsc70-4 are critical for clathrin-mediated endocytosis of silt diaphragm proteins.

### Slit diaphragm structure maintenance requires clathrin-mediated endocytosis

**Fig. 5:**
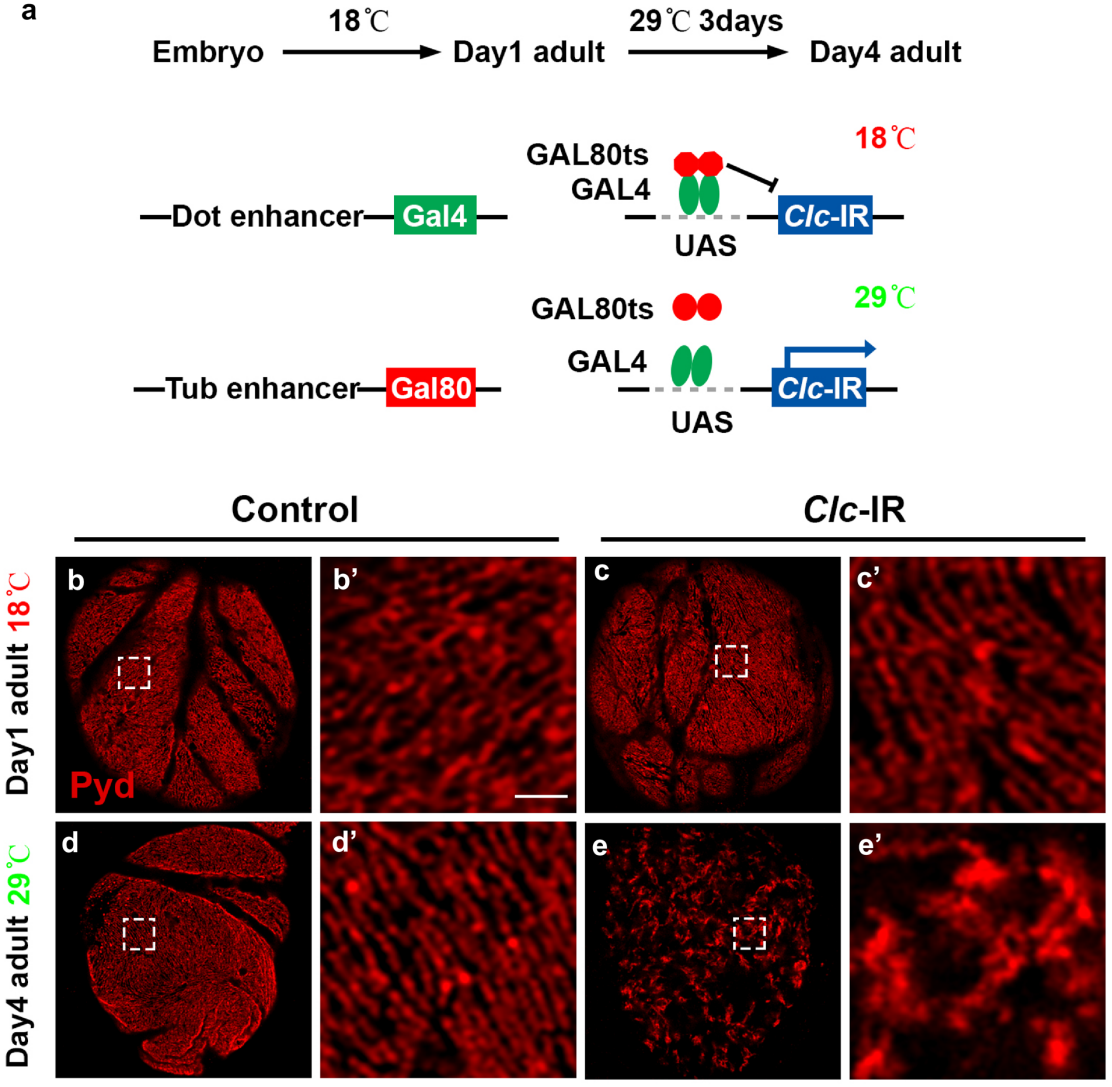
Nephrocyte slit diaphragm structure maintenance requires clathrin. **a** Schematic representation of the temperature sensitive *Gal80* experimental system. In brief, at 18 °C Gal80 is active, thus Gal80 inhibits Gal4 thereby preventing transcription. At 29 °C Gal80 no longer binds Gal4, thus Gal4 drives expression of *Clc*-IR thereby silencing expression of *Clc* specifically in the nephrocytes. *Dorothy* (*Dot*) promoter, nephrocyte-specific expression; *tubulin* (*tub*) promoter, ubiquitous expression pattern. **b**–**e** Immunofluorescence labeling for Pyd (red) of adult fly nephrocytes, visualized by confocal microscopy. Scale bar: 1 μm. **b**–**c′** At day one adult flies reared at 18 °C, wild type (Control; **b**–**b′**) and *Clc* RNAi knockdown (*Clc*-IR; **c**–**c′**) nephrocytes exhibited indistinguishable Pyd (red) distribution. In both, Pyd exhibited a uniform and smoothly distributed fingerprint-like pattern on the cell surface. **d**–**e′** At day four, after a three-day shift to 29 °C, nephrocyte cell surface Pyd distribution in wild type (Control) flies remained unchanged, showing the characteristic fingerprint-like pattern (**d**–**d′**). Nephrocytes in which the *Clc* gene was silenced (*Clc*-IR), displayed severe disruption of Pyd localization at the cell surface (**e**–**e′**)

The data above were obtained using a *Dot-Gal4* driver to silence the gene of interest. However, as this driver is already expressed early in fly development, the results might reflect the combined effects of early slit diaphragm formation during development and later maintenance after maturation. To test the requirement of CME in slit diaphragm maintenance specifically, we used the temperature sensitive *Gal80* to control the activity of *Dot-Gal4* (Fig. 5a). In this system, Gal80 activity can be controlled by the environmental temperature at which the flies are kept. *Gal80* is driven by the ubiquitous tubulin promoter and when expressed binds and represses the transcriptional activator *Gal4*. Fly embryos were kept at 18 °C (active Gal80 inhibits Gal4) until they eclosed as adult fly, allowing unimpeded maturation of the slit diaphragm structure. The one-day old flies were then transferred to 29 °C for 3 days (inactive Gal80, thus Gal4 drives expression of RNAi constructs), triggering target gene RNA knockdown (Fig. 5a). Using this system to control the knockdown of *Clathrin* (*Clc*), at 18 °C the localization of slit diaphragm protein (Pyd) in the nephrocytes of *Clc* transgenic flies was indistinguishable from that in the control group (Fig. 5b–c′). However, after shifting the flies to 29 °C for 3 days, the localization of slit diaphragm protein Pyd was severely disrupted (Fig. 5d–e′). The fingerprint-like pattern was replaced by disorganization and aggregation, highly similar to that observed with continuous *Clc* knockdown without *Gal80* (Fig. 1d–d″). These results suggest that after slit diaphragm maturation, CME pathway activity is required for slit diaphragm structure maintenance.

### Ultrastructural changes in nephrocytes with CME defects

**Fig. 6:**
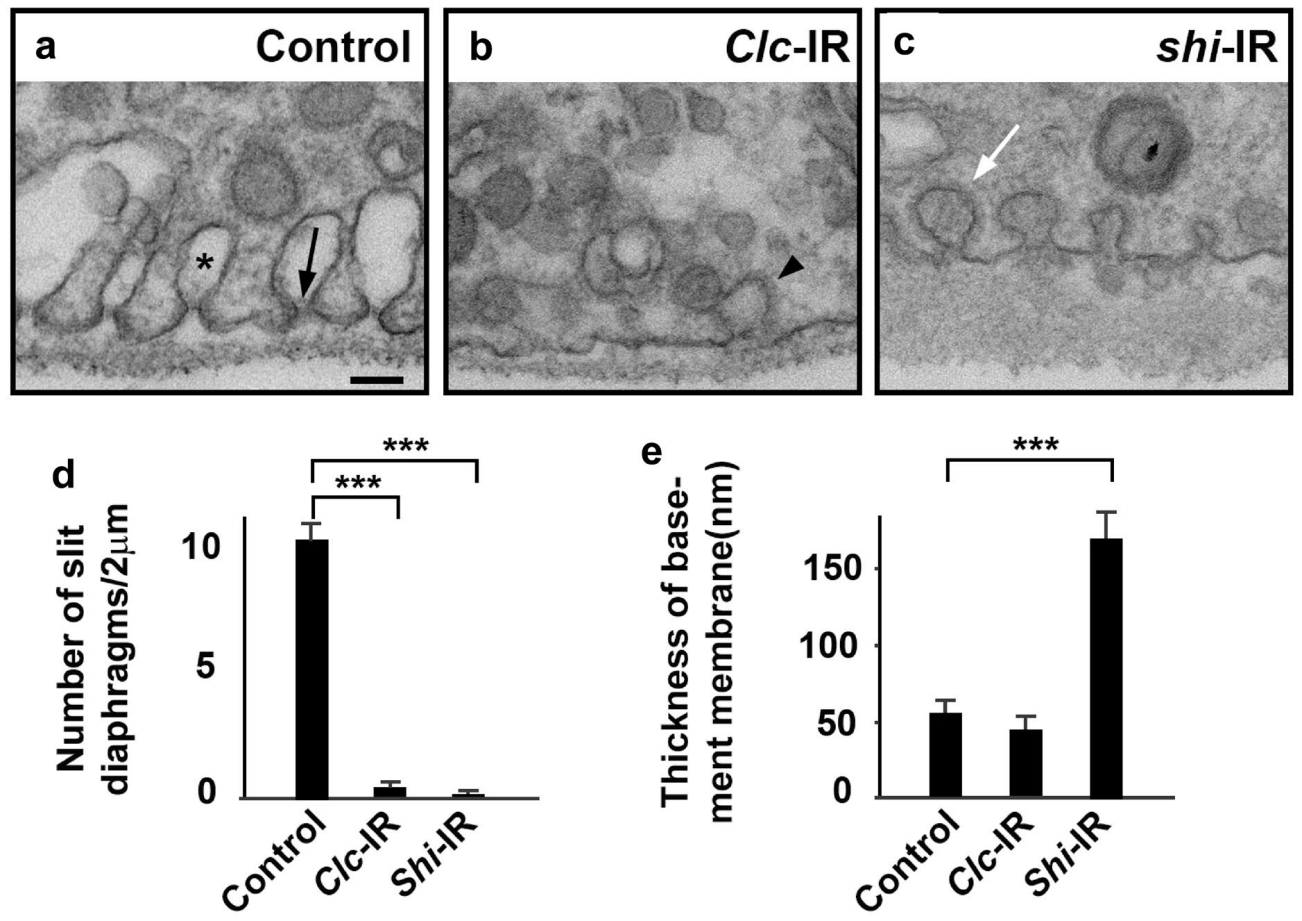
Ultrastructural changes in *Clc*- and *shi*-silenced nephrocytes. **a**–**c** Transmission electron microscopy (TEM) images of fly nephrocytes. Scale bar: 100 nm. **a** A wild type (Control) fly nephrocyte shows the typical characteristic ultrastructural features of regularly spaced slit diaphragms (black arrow) spanning the openings of lacuna channel membrane invaginations (asterisk). **b** *Clc* gene silencing (*Clc*-IR) eliminated the lacuna channels and slit diaphragm structures in the nephrocyte. Note the presence of many small vesicles as well as a vesicle undergoing endocytosis (arrowhead). **c** Slit diaphragm and lacuna channel structures were disrupted in *shi*-silenced (*shi*-IR) nephrocytes. The thickness of basement membrane increased significantly (see **e**). Note the presence of many Ω-shaped clathrin-coated pits at the cell membrane (white arrow). **d** Quantification of the number of slit diaphragms. In the TEM image, the cell membrane was divided into two 1 μm sections, then the number of slit diaphragms within each section was counted. At least seven sections were counted for each genotype. The results are presented as mean ± SD. Results were analyzed by Student’s t-test. ***P < 0.001. *Clc*-IR and *shi*-IR nephrocytes showed near total depletion of slit diaphragms. **e** Quantification of the thickness of the basement membrane. In the TEM image, the thickness of the basement membrane was measured in 1 μm intervals. At least ten measurements were obtained for each genotype. The results are presented as mean ± SD. Results were analyzed by Student’s t-test. ***P < 0.001. Membrane thickness in *Clc*-IR nephrocytes was unchanged when compared to wild type (Control). On the other hand, the basement membrane after silencing *shi* (*shi*-IR) was significantly thicker than that of wild type (Control) nephrocytes

To further establish the role of CME genes in regulating slit diaphragm structures, we examined the ultrastructural changes in *Clc*- and *shi*-silenced nephrocytes by TEM. In control nephrocytes, the slit diaphragm and lacuna channel structures are highly organized (Fig. 6a). In comparison, the number of slit diaphragm and lacuna channel structures in *Clc*-silenced nephrocytes were found to be greatly reduced (Fig. 6b, d). Although *Clc* depletion inhibits the CME pathway, both vesicles in the process of endocytosis as well as many small vesicles were visible in the *Clc*-silenced nephrocytes, thereby indicating the presence of a clathrin-independent endocytosis pathway in the nephrocytes. The CIE process also explains the accumulation of slit diaphragm proteins inside the cell in CME-gene-deficient nephrocytes, as these proteins were endocytosed through CIE pathway. The *shi*-silenced nephrocytes also showed severely decreased numbers of slit diaphragm and lacuna channel structures (Fig. 6c, d). We also frequently observed clathrin-coated pits which appeared unable to fission from the cell membrane in these nephrocytes, consistent with the role of Shi in the scission of clathrin-coated pits. Compared to *Clc*-silenced nephrocytes, there were much fewer vesicles in the *shi*-silenced nephrocytes, suggesting some vesicles in *Clc* depleted nephrocytes arose from clathrin-independent dynamin-dependent endocytosis. The thickness of basement membrane of *shi*-silenced nephrocyte increased dramatically, compared to both control wild type and *Clc*-silenced nephrocyte (Fig. 6e), which might be due to the accumulation of hemolymph proteins trapped in the basement membrane. This result suggests that some hemolymph proteins are cleared from the basement membrane through clathrin-independent but Shi (mammalian Dynamin)-dependent endocytosis. The ultrastructural changes in *Clc*- and *shi*-silenced nephrocytes are consistent with the role of CME genes in the maintenance of silt diaphragm structure, and also indicate the presence of a CIE pathway in nephrocytes.

### The endocytosed slit diaphragm proteins were recycled back to plasma membrane

**Fig. 7:**
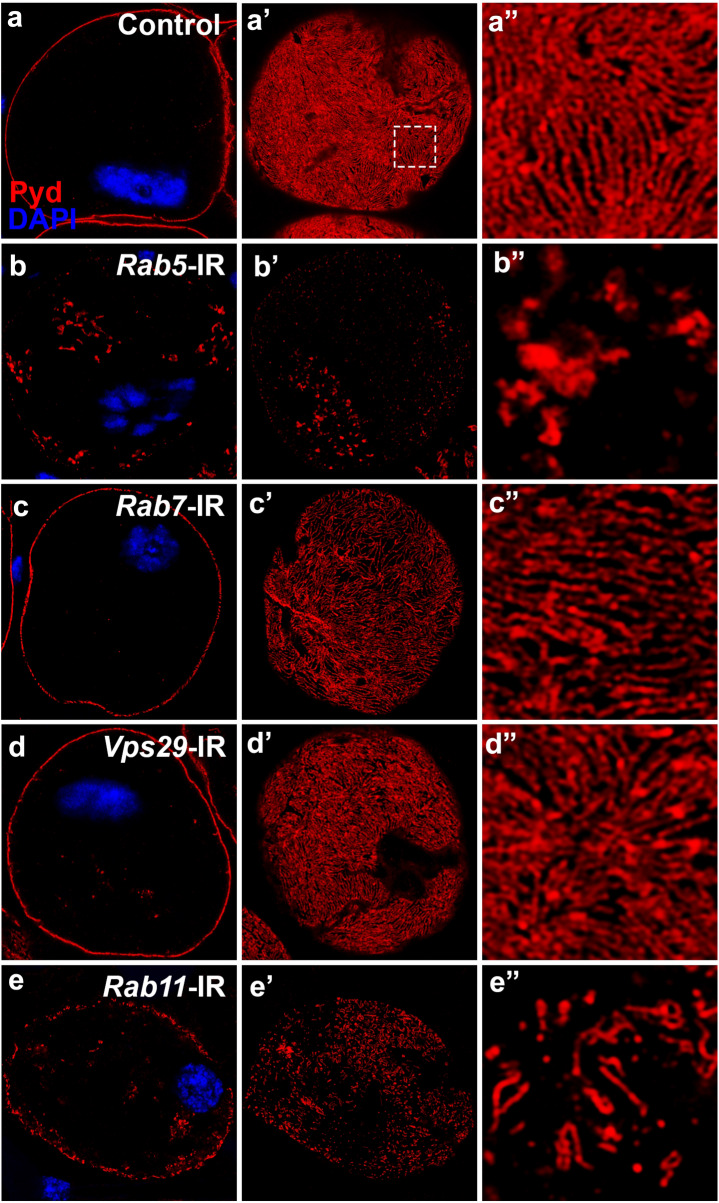
Genetic analysis of the destination of endocytosed slit diaphragm proteins. Immunofluorescence labeling for Pyd (red), with DAPI (blue) nuclear stain of adult fly nephrocytes, visualized by confocal microscopy. Scale bar: 1 μm. **a**–**a″** In wild type (Control) nephrocytes, Pyd was localized to the cell membrane (**a**) and exhibited a uniform and smoothly distributed fingerprint-like pattern on the cell surface (**a′**–**a″**). **b**–**b″** and **e**–**e″** Silencing of *rab5* and *rab11* resulted in intracellular aggregation of Pyd proteins and disruption of Pyd cell surface localization. Rab5 is a critical regulator of early endosome formation, while Rab11 is important for recycling endosome function. **c**–**d** Silencing of *rab7* (**c**–**c″**) or *vps29* (**d**–**d″**) did not disrupt Pyd cell surface localization. Rab7 is critical for late endosome maturation, and Vps29 is a component of the Retromer complex, which is required for early endosome to Golgi retrograde trafficking

Vesicles with cargo proteins endocytosed through CME pathway would fuse with early endosomes, the formation of which is controlled by Rab5 GTPase [18]. For most the cargo proteins in the early endosomes, they were sorted into three destinations: the late endosome through Rab7 GTPase, the Golgi through the Retromer complex and the recycling endosome through Rab11 GTPase [18]. We screened genes involved in these processes (Additional file 1: Table S1) and found that silencing Rab5 or Rab11 lead to mislocalization of Pyd protein similar to CME pathway genes, while silencing Rab7 or the Retromer did not obviously alter the localization of Pyd protein (Fig. 7 and data not shown), suggesting Rab5-dependent early endosome and Rab11-dependent recycling endosome are required for slit diaphragm protein endocytosis and recycling. The recycling of some cargo proteins back to plasma membrane does not require Rab11, but may depend on other Rab GTPases like Rab4, Rab10 or Rab35. We also tested these Rabs and found that they were not required for slit diaphragm protein Pyd recycling (data not shown). Our results indicate the slit diaphragm proteins endocytosed by CME pathway were accepted by the early endosome, and then sorted into recycling endosome, with plasma membrane as the final destination.

## Discussion

Maintaining the integrity of the slit diaphragm structure is essential for the glomerular filtration function, as illustrated by many nephrotic mutations affecting slit diaphragm proteins. Nephrin has been proposed to undergo clathrin-mediated endocytosis in cultured cells or in mice podocyte in vivo [22, 23, 33]. However, questions remained regarding the role of the CME pathway in regulating the slit diaphragm structure. The data presented here, clearly demonstrate that the clathrin-mediated endocytosis pathway plays a critical role in maintaining the nephrocyte slit diaphragm structure. As components of the CME pathway are highly conserved between fly and human (Additional file 1: Table S1), our results suggest a similar role for CME components in regulating the slit diaphragm structure in human podocytes.

**Fig. 8:**
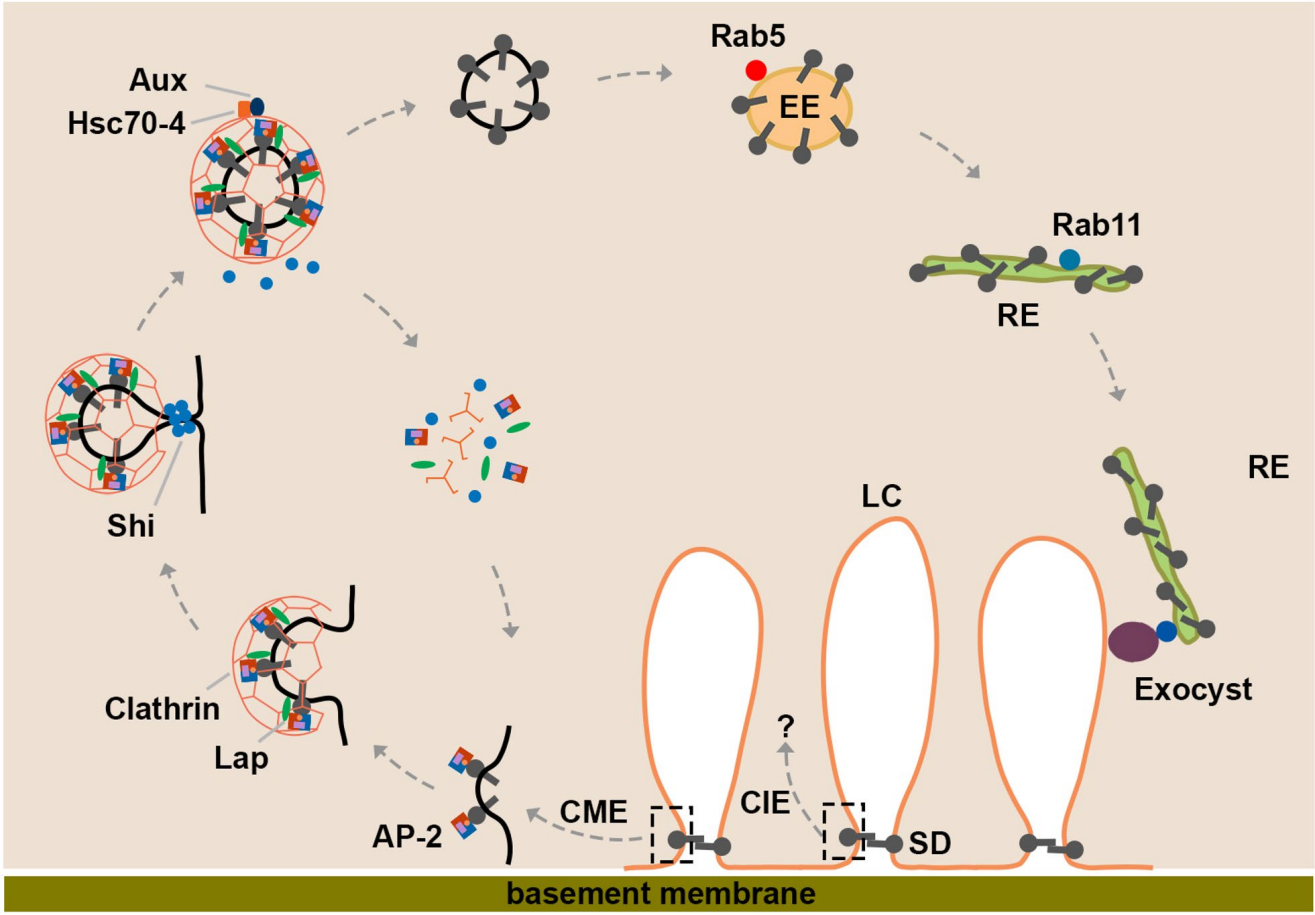
Schematic representation of slit diaphragm protein endocytosis and recycling pathway. In the *Drosophila* nephrocyte, plasma membrane invaginates to form lacuna channels (LC). Slit diaphragm (SD) forms at the mouth of these invaginations. The left dashed box indicates the process of slit diaphragm protein endocytosis through the clathrin-mediated endocytosis (CME) pathway. The slit diaphragm proteins at the cell membrane are recognized by the AP-2 complex or other adaptor proteins. Then the AP-2 complex recruits the clathrin complex and like-AP180 (Lap) adaptor to assemble the clathrin coat. Subsequently Shibire (Shi) mediates fission of the endocytic pit from the cell membrane. The clathrin-coated vesicles are later disassembled by auxilin (Aux) and Hsc70-4, releasing AP-2, clathrin and Shi for the next cycle of endocytosis. The uncoated vesicles enter the Rab5-positive early endosome (EE). The slit diaphragm protein cargo is sorted into the Rab11-dependent recycling endosome (RE). Rab11 recruits the exocyst complex to tether the recycling endosome to the cell membrane. The two membranes fuse and the slit diaphragm proteins recycle back to the cell membrane to facilitate assembly of the slit diaphragm structure. The right dashed box indicates slit diaphragm protein endocytosis through the clathrin-independent endocytosis (CIE) pathway, the exact route of which is not clear, which is indicated by the question mark

Silencing genes in the CME pathway led to defective endocytosis of slit diaphragm protein, disruption of the slit diaphragm structure and reduced nephrocyte function. These phenotypes are very similar to those we reported previously for nephrocytes in which *Rab5*, *Rab11* or genes of the exocyst complex were silenced [35]. Based on those and our current results, we propose the following process of endocytosis and recycling for slit diaphragm proteins (Fig. 8). First, the slit diaphragm proteins on the cell membrane are recognized by the AP-2 complex or other adaptor proteins. Then the AP-2 complex recruits the clathrin complex and additional adaptors, like Lap, to assemble the clathrin coat. Protein Shi mediates fission of these endocytic pits from the cell membrane. The clathrin-coated vesicles are later disassembled by Aux and Hsc70-4, thereby releasing clathrin, AP-2, Lap and Shi for the next cycle of endocytosis. The uncoated vesicles then enter the early endosome, formation of which requires Rab5. Next the slit diaphragm protein cargo is sorted into the Rab11-dependent recycling endosome. Rab11 recruits the exocyst complex to tether the recycling endosome to cell membrane. Finally, the two membranes fuse and the slit diaphragm proteins recycle back to the cell membrane to facilitate assembly of the slit diaphragm structure. There are still important questions left to be addressed: What triggers endocytosis of slit diaphragm proteins? How are the slit diaphragm cargo proteins selectively sorted into the Rab11-dependent recycling endosome instead of other destinations?

Interestingly, *Clathrin* (*Clc*)-silenced nephrocytes still had vesicles undergoing endocytosis, with many vesicles visible inside the cell (Fig. 6b). In addition, slit diaphragm proteins accumulated in the cytoplasm in nephrocytes with depleted CME pathway genes. If slit diaphragm proteins can be endocytosed only through the CME pathway, these proteins would have accumulated on the cell membrane, instead of being trapped in the cytoplasm. Therefore, we reason that a clathrin-independent endocytosis (CIE) pathway has to be present to transport slit diaphagm proteins into the cytoplasm when the CME pathway is blocked. Slit Diaphgam (SD) proteins could enter the cytoplasm through both CME and CIE, but can only be recycled back to the nephrocyte cell membrane through the CME pathway. Therefore, we speculate that a protein quality check process is present in the CME pathway (but not in the CIE pathway) to ensure non-damaged SD proteins to be recycled. We are currently investigating this protein quality check process in the CME pathway. We are also searching for the CIE pathway that is involved in slit diaphragm endocytosis, and try to identify its components and biological function.

Our results demonstrate that the *Drosophila* nephrocyte makes an excellent model system to identify genes involved in slit diaphragm protein endocytosis and recycling. In fact, the nephrocyte can also serve as an effective model to study general endocytosis and recycling. The human homologs for the CME genes which we identified in this study should be examined for possible mutations in patients with nephrotic syndrome. We could target the regulators in the CME pathway to improve the function of podocytes and find better treatment for patients with nephrotic syndrome. For example, Bis-T-23, a drug that increases the activity of dynamin, was able to ameliorate glomerular damage in multiple vertebrate models of kidney diseases [24]. The *Drosophila* nephrocyte model provides a versatile platform for identifying additional candidate genes in glomerular disease, to unravel the molecular mechanism of glomerular dysfunction and uncover potential pharmacological interventions for glomerular disease.

## Materials and methods

### Fly strains

All fly stocks were kept on standard food at 25 °C. Flies carrying *Hand-GFP*, *Dot-Gal4*, *MHC-ANF-RFP* transgenes have been described previously [36]. The *Dot-Gal4* (#67608) and all *UAS*-RNAi transgenic RNAi lines were obtained from the Bloomington Drosophila stock center: *UAS-shi*^RNAi^ (#28513), *UAS-Chc*^RNAi^ (#27530), *UAS-Clc*^RNAi^ (#27496), *UAS-Arf79F*^RNAi^ (#66174), *UAS-AP-2α*^RNAi^ (#27322), *UAS-AP-2μ*^RNAi^ (#28040), *UAS-AP-2σ*^RNAi^ (#27322), *UAS-AP-1-2β*^RNAi^ (#28328), *UAS-AP-1γ*^RNAi^ (#27533), *UAS-AP-1μ*^RNAi^ (#27534), *UAS-AP-1σ*^RNAi^ (#40895), *UAS-lap*^RNAi^ (#28358), *UAS-aux*^RNAi^ (#28509), *UAS-Hsc70-4*^RNAi^ (#28709), *UAS-Rab5*^RNAi^ (#34832), *UAS-Rab7*^RNAi^ (#27051), *UAS-Vps29*^RNAi^ (#38963), *UAS-Rab11*^RNAi^ (#42709).

### ANF-RFP uptake assay

Briefly, 10 virgin female flies from the *MHC-ANF-RFP*, *Hand-GFP* and Dot-Gal4 transgenic lines were crossed to 5 male flies from *UAS*-RNAi transgenic lines at 25 °C. Pericardial nephrocytes of newly emerged adult flies (within 24 h of eclosion) were dissected and kept in *Drosophila* artificial hemolymph to assay RFP accumulation detected by fluorescence microscopy.

### Dextran uptake assay

Flies carrying *Hand-GFP* and *Dot-Gal4* transgenes were crossed with flies carrying the *UAS*-RNAi transgenes at 25 °C. Dextran uptake was assessed in adult flies one-day post-emergence by dissection of pericardial nephrocyte in *Drosophila* artificial hemolymph and examination of the cells by fluorescence microscopy after a 20 min incubation with Texas Red labeled Dextran (10 kD, 0.02 mg/ml).

### Immunohistochemistry

Pericardial nephrocytes from one day post-emergence adult flies was dissected in *Drosophila* artificial hemolymph and then heat-fixed for 15 s in *Drosophila* artificial hemolymph preheated to 103 °C. Tissues were washed twice for 10 min with 1X PBST (1X phosphate buffered saline (PBS), with 0.1% TritonX-100). Tissues were then blocked with 2% BSA (Bovine Serum Albumin) in 1X PBST for 30 min. Samples were incubated with primary antibody overnight at 4 °C. Tissues were washed three times for 10 min with 1X PBST and then incubated with secondary antibody for 2 h at room temperature. Tissues were washed again thrice for 10 min with 1X PBST and mounted using Vectashield mounting media. The mouse monoclonal anti-Pyd antibody (PYD2) was obtained from Developmental Studies Hybridoma Bank. The rabbit anti-Sns antibody was a gift from Dr. Britta George [2]. The rabbit anti-Clc antibody was kindly provided by Dr. Graeme Davis [8]. The rabbit anti-Rab7 antibody was from Dr. Akira Nakamura. The Alexa Fluor-conjugated secondary antibodies were ordered from Thermo Fisher Scientific.

### Confocal imaging

Confocal imaging was performed with a Zeiss LSM900 microscope using a 63x Plan-Apochromat 1.4 N.A. oil objective under Airyscan SR mode. For quantitative comparisons of fluorescence intensity, common settings were chosen to avoid oversaturation. ImageJ Software was used for image processing.

### Transmission electron microscopy (TEM)

TEM was carried out using standard procedures. Briefly, one-day-old adult flies of the indicated genotypes were dissected in artificial hemolymph and fixed in 8% paraformaldehyde for 10 min. Then the tissues were further trimmed in 1X PBS. The trimmed samples were transferred into fixation buffer containing 4% paraformaldehyde and 2.5% glutaraldehyde. The samples were further processed and analyzed using a FEI Tecnai T12 TEM.

### Statistics

Statistical analyses were performed with Graphpad Prism 7 software. For quantification of relative ANF-RFP or Dextran-linked Texas Red fluorescence, 20 nephrocytes were analyzed from each of 3 flies per indicated genotype. For quantification of the thickness of basement membrane, 10 measurements were analyzed in each genotype. Each data set is presented as mean ± SD. Results were analyzed by two-tailed Student’s t-test. P values < 0.05 were considered statistically significant (**P < 0.01; ***P < 0.001).

## Supplementary Information

**Additional file1: Table S1**. The RNAi screen of endocytosis genes required for slit diaphragm protein endocytosis and recycling. **Figure S1**. The clathrin genes, Chc and Clc, are required for nephrocyte function. **A** MHC-ANF-RFP derived hemolymph ANF-RFP (red) uptake by nephrocytes. Hand-GFP labels both nephrocytes (big nucleus) and heart cells (small nucleus). Wild type (Control) flies accumulated abundant ANF-RFP. Subsequent panels show that Chc and Clc genes RNAi silencing severely reduced the ANF-RFP accumulation level. **B** Texas Red-labeled 10 kD Dextran particles uptake by nephrocytes. Particles were easily taken in and collected by wild type control nephrocytes in an ex vivo assay. Silencing of Chc and Clc genes clearly reduced levels of accumulated Dextran. **C** Quantification of relative ANF-RFP fluorescence in nephrocytes expressing the indicated gene silencing RNAi construct. **D** Quantification of relative Dextran-linked Texas Red fluorescence in nephrocytes expressing the indicated gene silencing RNAi construct. ***P<0.001. **Figure S2**. The clathrin genes, Chc and Clc, are required for slit diaphragm protein Sns endocytosis. **A-A’** Sns (red) distributed uniformly and smoothly in parallel lines in fingerprint like pattern in control (wild type) nephrocytes. Scale bar: 1μm. **B-C’** Silencing of Chc and Clc resulted in severe disruption of Sns cell surface localization. The Sns-positive lines became more curved and shorter, and many became dots. The lines were no longer parallel, and the spacing became irregular. **Figure S3**. The *Drosophila* dynamin homolog, Shi, is required for nephrocyte function. **A** MHC-ANF-RFP derived hemolymph ANF-RFP (red) uptake by nephrocytes. Hand-GFP labels both nephrocytes (big nucleus) and heart cells (small nucleus). Wild type (Control) flies accumulated abundant ANF-RFP. Subsequent panel shows that *shi* gene RNAi silencing severely reduced the ANF-RFP accumulation level. **B** Quantification of relative ANF-RFP fluorescence in nephrocytes expressing the indicated gene silencing RNAi construct. **C** Texas Red-labeled 10 kD Dextran particles uptake by nephrocytes. Particles were easily taken in and collected by wild type control nephrocytes in an ex vivo assay. Silencing *shi* clearly reduced levels of accumulated Dextran. **D** Quantification of relative Dextran-linked Texas Red fluorescence in nephrocytes expressing the indicated gene silencing RNAi construct. ***P<0.001. **Figure S4**. The *Clc*-IR transgene can specifically decrease Clc protein level. Anti-Clc antibody fluorescent staining of Clc expression (red) in nephrocytes. Left panel, in control nephrocytes, Clc is strongly expressed and localized mainly close to plasma membrane. Right panel, in *clc*-silenced nephrocytes, the protein level of Clc is below the detection limit, suggesting highly effective knockdown. **Figure S5**. AP-2 complex is required for nephrocyte function. **A** MHC-ANF-RFP derived hemolymph ANF-RFP (red) uptake by nephrocytes. Hand-GFP labels both nephrocytes (big nucleus) and heart cells (small nucleus). Wild type (Control) flies accumulated abundant ANF-RFP. Subsequent panels show that AP-2α, AP-2μ, AP-2Ω and AP-1-2β genes RNAi silencing severely reduced the ANF-RFP accumulation level. **B** Quantification of relative ANF-RFP fluorescence in nephrocytes expressing the indicated gene silencing RNAi construct. **C** Texas Red-labeled 10 kD Dextran particles uptake by nephrocytes. Particles were easily taken in and collected by wild type control nephrocytes in an ex vivo assay. Silencing of AP-2α, AP-2μ, AP-2Ω and AP-1-2β genes clearly reduced levels of accumulated Dextran. **D **Quantification of relative Dextran-linked Texas Red fluorescence in nephrocytes expressing the indicated gene silencing RNAi construct. ***P<0.001. **Figure S6**. AP-2 complex is required for Sns protein proper localization. **A-A’** Sns (red) distributed uniformly and smoothly in parallel lines in finger-print like pattern in control (wild type) nephrocytes. Scale bar: 1μm. **B-D** Silencing of AP-1-2β, AP-2α, AP-2μ and AP-2σ resulted in severe disruption of Sns cell surface localization. **Figure S7**. AP-1 complex is not required for Pyd protein proper localization. **A-A″** Sns (red) distributed uniformly and smoothly in parallel lines in finger-print like pattern in control (wild type) nephrocytes, with DAPI (blue nuclear stain). Scale bar: 1μm. **B**-**D″** Silencing of AP-1γ, AP-1σ and AP-1μ showed similar pattern of Pyd cell surface localization as the control. **Figure** **S8**. The adaptor protein Lap and uncoating proteins, Aux and Hsc70-4, are required for nephrocyte function. **A** MHC-ANF-RFP derived hemolymph ANF-RFP (red) uptake by nephrocytes. Hand-GFP labels both nephrocytes (big nucleus) and heart cells (small nucleus). Wild type (Control) flies accumulated abundant ANF-RFP. Subsequent panels show that Lap, Aux and Hsc70-4 genes RNAi silencing severely reduced the ANF-RFP accumulation level. **B** Quantification of relative ANF-RFP fluorescence in nephrocytes expressing the indicated gene silencing RNAi construct. **C** Texas Red-labeled 10 kD Dextran particles uptake by nephrocytes. Particles were easily taken in and collected by wild type control nephrocytes in an ex vivo assay. Silencing of Lap, Aux and Hsc70-4 genes clearly reduced levels of accumulated Dextran. **D** Quantification of relative Dextran-linked Texas Red fluorescence in nephrocytes expressing the indicated gene silencing RNAi construct. ***P<0.001. **Figure S9**. The adaptor protein Lap and uncoating proteins, Aux and Hsc70-4, are required for Sns protein proper localization. **A-A’** Sns (red) distributed uniformly and smoothly in parallel lines in fingerprint like pattern in control (wild type) nephrocytes. Scale bar: 1μm. **B**-**D’** Silencing of Lap, Aux, Hsc70-4 resulted in severe disruption of Sns cell surface localization. **Figure S10**. The *Rab7*-IR transgene can specifically decrease Rab7 protein level. Anti-Rab7 antibody fluorescent staining of Rab7 expression (green) in nephrocytes, with DAPI (blue) nuclear stain. In control nephrocytes, Rab7 is strongly expressed. In *rab7*-silenced nephrocytes, the protein level of Rab7 is significantly reduced, suggesting highly effective knockdown.

## Data Availability

All data and materials generated in this study are available publicly upon request.
