# Supplementary material for: Slit diaphragm maintenance requires dynamic clathrin-mediated endocytosis facilitated by AP-2, Lap, Aux and Hsc70-4 in nephrocytes
Source: Cell Biosci. 2021 May 11;11:83. doi: 10.1186/s13578-021-00595-4 (PMC8111712; doi:10.1186/s13578-021-00595-4)

Supplemental Table 1:

Systematic screen of endocytic genes for NSD maintenance

Drosophila Genes	Human homologues	DIOPT score	Function	Slit diaphragm strucutre
Clc	CLTA	13	Clathrin-mediated endocytosis	Structure disrupted
Chc	CLTC	14		
AP-1-2β	AP2B1	12		
AP-2α	AP2A2	13		
AP-2μ	AP2M1	13		
AP-2σ	AP2S1	14		
Shi	DYNAMIN1	13		
Aux	AUXILIN	11		
Hsc70-4	HSPA8	12		
Lap	AP180	12		
Rab5	RAB5B	13	Early endosome	Structure disrupted
Rbsn-5	RBSN	11		
Rab7	RAB7A	13	Late endosome	Normal
Vps29	VPS29	14	Retromer	Normal
Vps26	VPS26A	12		
Vps35	VPS35	14		
Rab11	RAB11A	13	Recycling endosome	Structure disrupted
Sec5	EXOC2	14	Exocyst	Structure disrupted
Sec6	EXOC3	14		
Sec10	EXOC5	14		
Sec15	EXOC6	13		
Exo84	EXOC8	14		
AP-1γ	AP1G1	13	AP-1	Normal
AP-1μ	AP1M1	14		
AP-1σ	AP1S2	12		
Arf79F	ARF1	12	Clathrin-independent endocytosis	Reduced density

Gene that are required for Nephrocyte Slit Diaphragm (NSD) maintenance are highlighted. DIOPT scores (all >10) indicate that these genes are highly conserved from flies to humans.

Supplemental Figure Legends

Supplemental Table 1: **The RNAi screen of endocytosis genes required for slit diaphragm protein endocytosis and recycling.** There are usually multiple human genes for the fly homolog and here only showed the representative one. DIOPT score is calculated by the DRSC Integrative Ortholog Prediction Tool (DIOPT) at http://www.flyrnai.org/diopt. The max score between fly and human homologs is 16, and genes with a score higher than 2 could be considered as orthologs.

Supplemental Figure 1: **The clathrin genes, Chc and Clc, are required for nephrocyte function.** **A** MHC-ANF-RFP derived hemolymph ANF-RFP (red) uptake by nephrocytes. Hand-GFP labels both nephrocytes (big nucleus) and heart cells (small nucleus). Wild type (Control) flies accumulated abundant ANF-RFP. Subsequent panels show that Chc and Clc genes RNAi silencing severely reduced the ANF-RFP accumulation level. **B** Quantification of relative ANF-RFP fluorescence in nephrocytes expressing the indicated gene silencing RNAi construct. **C** Texas Red-labeled 10 kD Dextran particles uptake by nephrocytes. Particles were easily taken in and collected by wild type control nephrocytes in an ex vivo assay. Silencing of Chc and Clc genes clearly reduced levels of accumulated Dextran. **D** Quantification of relative Dextran-linked Texas Red fluorescence in nephrocytes expressing the indicated gene silencing RNAi construct. ***P<0.001.

Supplemental Figure 2: **The clathrin genes, Chc and Clc, are required for slit diaphragm protein Sns endocytosis. A-A'** Sns (red) distributed uniformly and smoothly in parallel lines in finger-print like pattern in control (wild type) nephrocytes. Scale bar: 1μm. **B-H** Silencing of Chc and Clc resulted in severe disruption of Sns cell surface localization. The Sns-positive lines became more curved and shorter, and many became dots. The lines were no longer parallel, and the spacing became irregular.

Supplemental Figure 3: **The Drosophila dynamin homolog, Shi, is required for nephrocyte function. A** MHC-ANF-RFP derived hemolymph ANF-RFP (red) uptake by nephrocytes. Hand-GFP labels both nephrocytes (big nucleus) and heart cells (small nucleus). Wild type (Control) flies accumulated abundant ANF-RFP. Subsequent panels show that Chc and Clc genes RNAi silencing severely reduced the ANF-RFP accumulation level. **B** Quantification of relative ANF-RFP fluorescence in nephrocytes expressing the indicated gene silencing RNAi construct. **C** Texas Red-labeled 10 kD Dextran particles uptake by nephrocytes. Particles were easily taken in and collected by wild type control nephrocytes in an ex vivo assay. Silencing of Chc and Clc genes clearly reduced levels of accumulated Dextran. **D** Quantification of relative Dextran-linked Texas Red fluorescence in nephrocytes expressing the indicated gene silencing RNAi construct. ***P<0.001.

Supplemental Figure 4: **The *Clc*-IR transgene can specifically decrease Clc protein level.** Anti-Clc antibody fluorescent staining of Clc expression (red) in nephrocytes. **A** In control nephrocytes, Clc is strongly expressed and localized mainly close to plasma membrane. **B** In *clc*-silenced

nephrocytes, the protein level of Clc is below the detection limit, suggesting highly effective knockdown.

Supplemental Figure 5: **AP-2 complex is required for nephrocyte function. A** MHC-ANF-RFP derived hemolymph ANF-RFP (red) uptake by nephrocytes. Hand-GFP labels both nephrocytes (big nucleus) and heart cells (small nucleus). Wild type (Control) flies accumulated abundant ANF-RFP. Subsequent panels show that Chc and Clc genes RNAi silencing severely reduced the ANF-RFP accumulation level. **B** Quantification of relative ANF-RFP fluorescence in nephrocytes expressing the indicated gene silencing RNAi construct. **C** Texas Red-labeled 10 kD Dextran particles uptake by nephrocytes. Particles were easily taken in and collected by wild type control nephrocytes in an ex vivo assay. Silencing of Chc and Clc genes clearly reduced levels of accumulated Dextran. **D** Quantification of relative Dextran-linked Texas Red fluorescence in nephrocytes expressing the indicated gene silencing RNAi construct. ***$P<0.001$.

Supplemental Figure 6: **AP-2 complex is required for Sns protein proper localization. A-A'** Sns (red) distributed uniformly and smoothly in parallel lines in finger-print like pattern in control (wild type) nephrocytes. Scale bar: 1μm. **B-D** Silencing of AP-2α, AP-2μ and AP-2σ resulted in severe disruption of Sns cell surface localization.

Supplemental Figure 7: **AP-1 complex is not required for Pyd protein proper localization. A-A'** Sns (red) distributed uniformly and smoothly in parallel lines in finger-print like pattern in control (wild type) nephrocytes. Scale bar: 1μm. **B-D** Silencing of AP-1γ, AP-1σ and AP-1μ showed similar pattern of Pyd cell surface localization as the control.

Supplemental Figure 8: **The adaptor protein Lap and uncoating proteins, Aux and Hsc70-4, are required for nephrocyte function. A** MHC-ANF-RFP derived hemolymph ANF-RFP (red) uptake by nephrocytes. Hand-GFP labels both nephrocytes (big nucleus) and heart cells (small nucleus). Wild type (Control) flies accumulated abundant ANF-RFP. Subsequent panels show that Aux and Hsc70-4 genes RNAi silencing severely reduced the ANF-RFP accumulation level. **B** Texas Red-labeled 10 kD Dextran particles uptake by nephrocytes. Particles were easily taken in and collected by wild type control nephrocytes in an ex vivo assay. Silencing of Aux and Hsc70-4 genes clearly reduced levels of accumulated Dextran. **C** Quantification of relative ANF-RFP fluorescence in nephrocytes expressing the indicated gene silencing RNAi construct. **D** Quantification of relative Dextran-linked Texas Red fluorescence in nephrocytes expressing the indicated gene silencing RNAi construct. ***$P<0.001$.

Supplemental Figure 9: **The uncoating proteins, Aux and Hsc70-4, are required for Sns protein proper localization. A-A'** Sns (red) distributed uniformly and smoothly in parallel lines in finger-print like pattern in control (wild type) nephrocytes. Scale bar: 1μm. **B-C** Silencing of Aux, Hsc70-4 resulted in severe disruption of Sns cell surface localization.

Supplemental Figure 10: **The *Rab7*-IR transgene can specifically decrease Clc protein level.** Anti-Rab7 antibody fluorescent staining of Rab7 expression (green) in nephrocytes. In control

nephrocytes, Rab7 is strongly expressed. In *rab7*-silenced nephrocytes, the protein level of Rab7 is significantly reduced, suggesting highly effective knockdown

Supplemental Figure 1

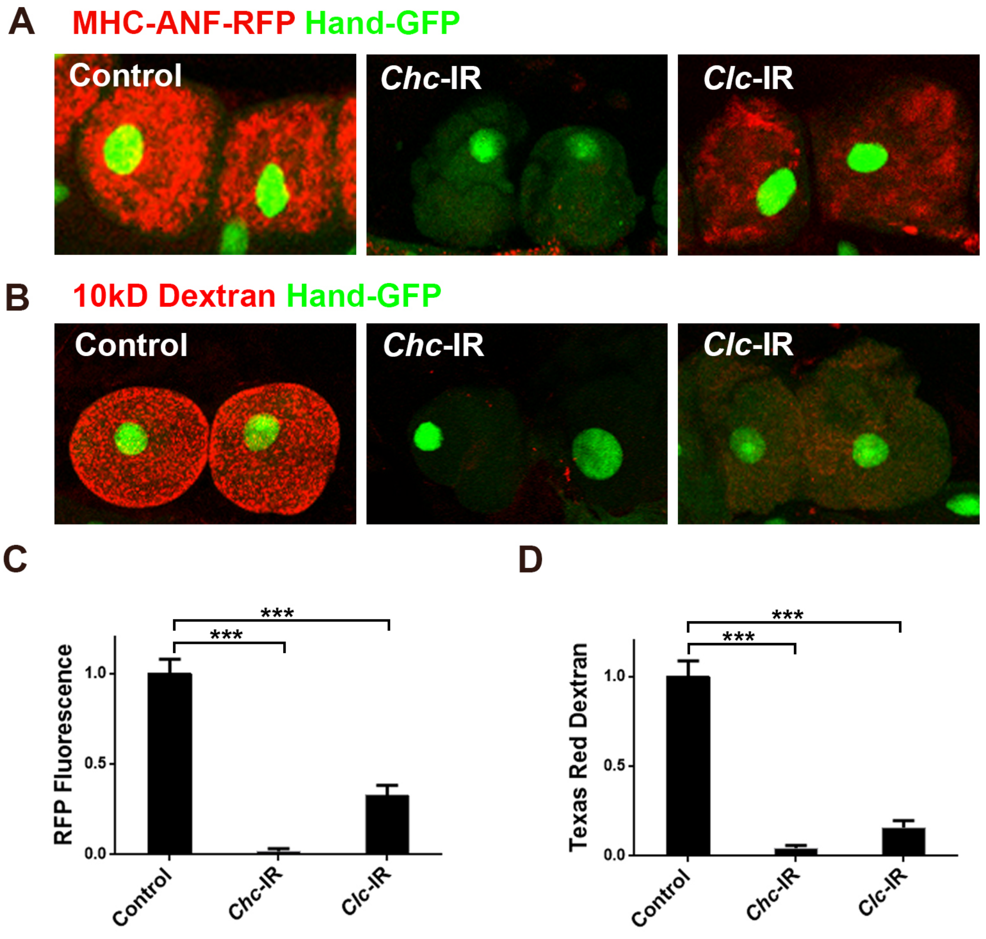

Supplemental Figure 2

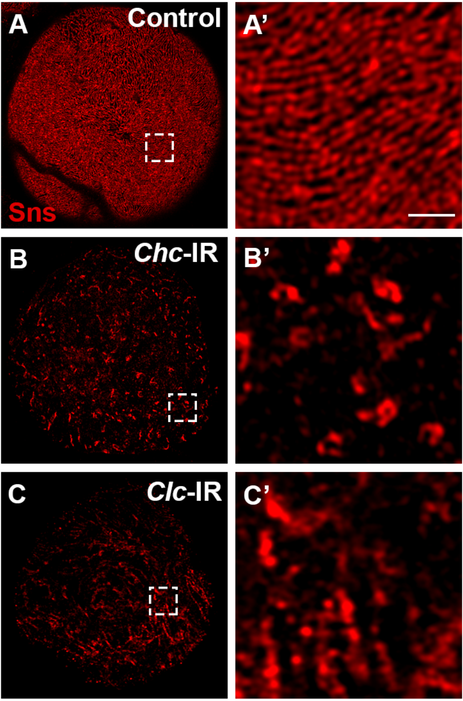

Supplemental Figure 3

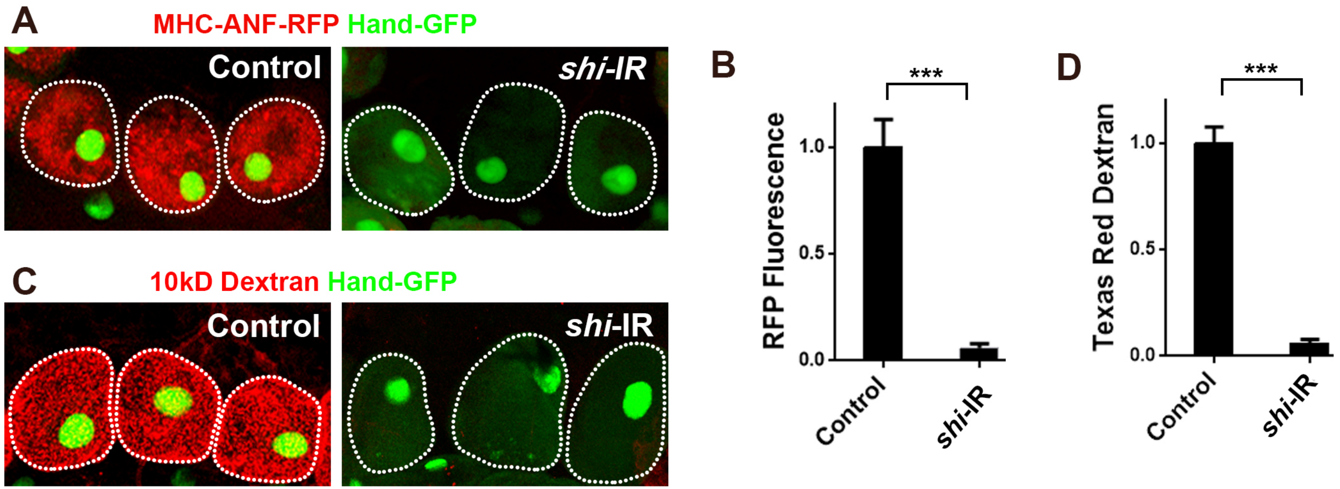

Supplemental Figure 4

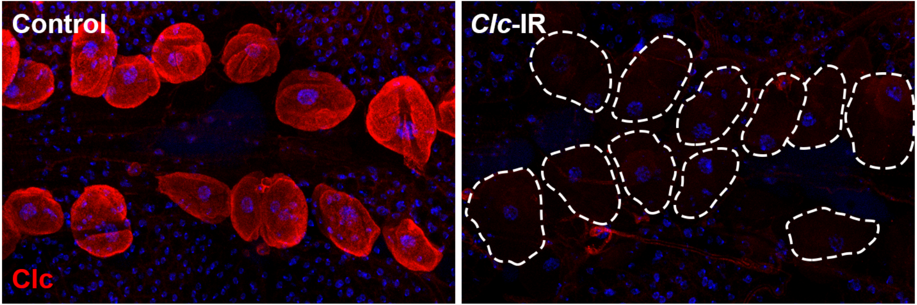

Supplemental Figure 5

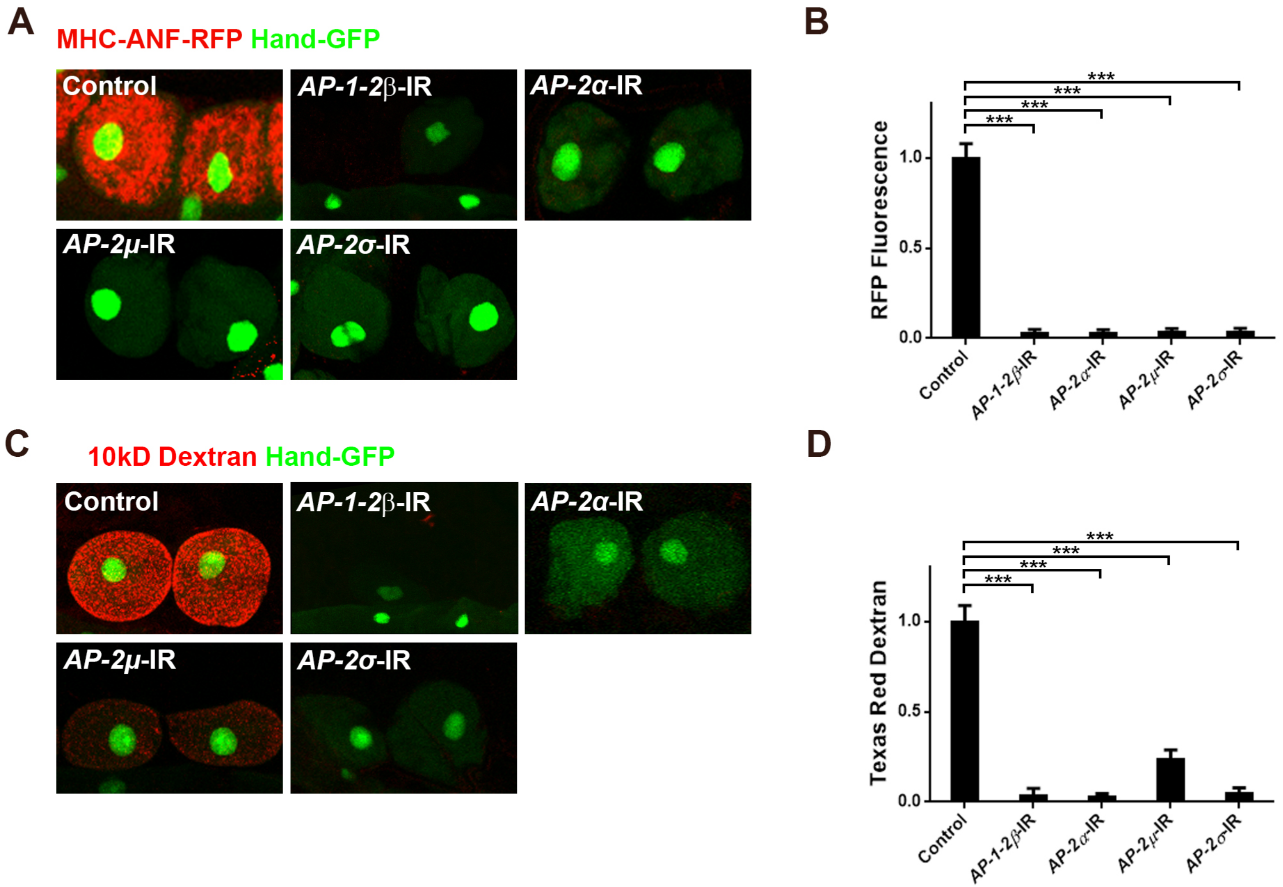

Supplemental Figure 6

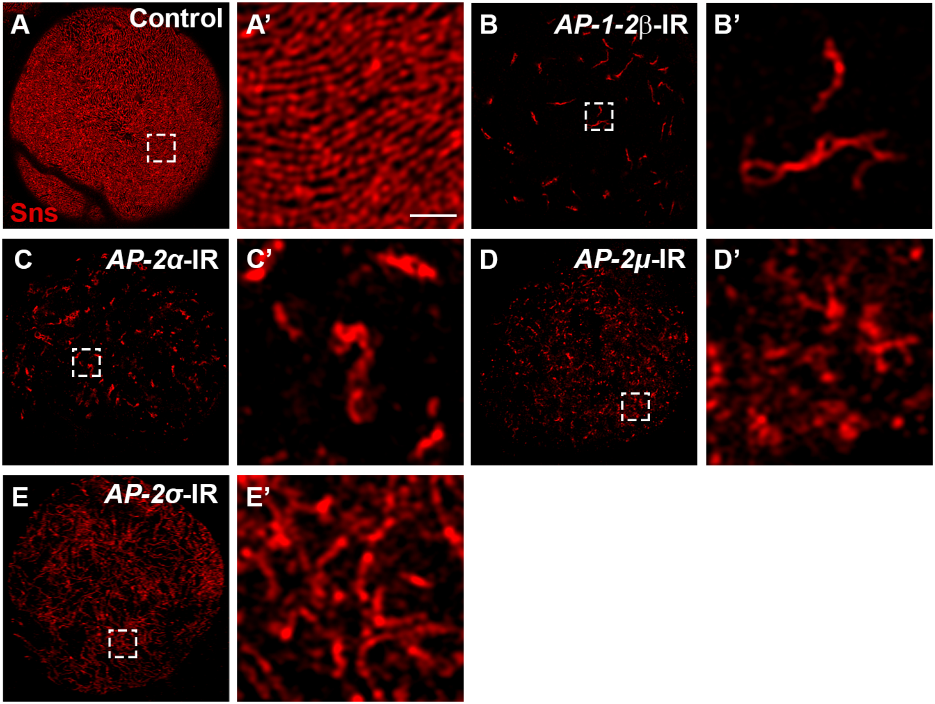

Supplemental Figure 7

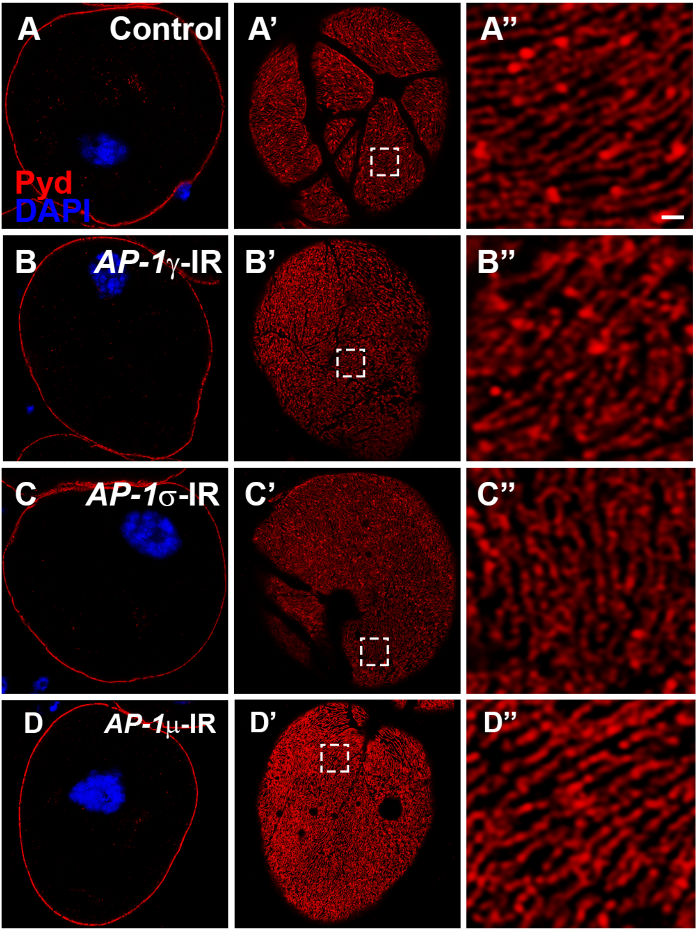

Supplemental Figure 8

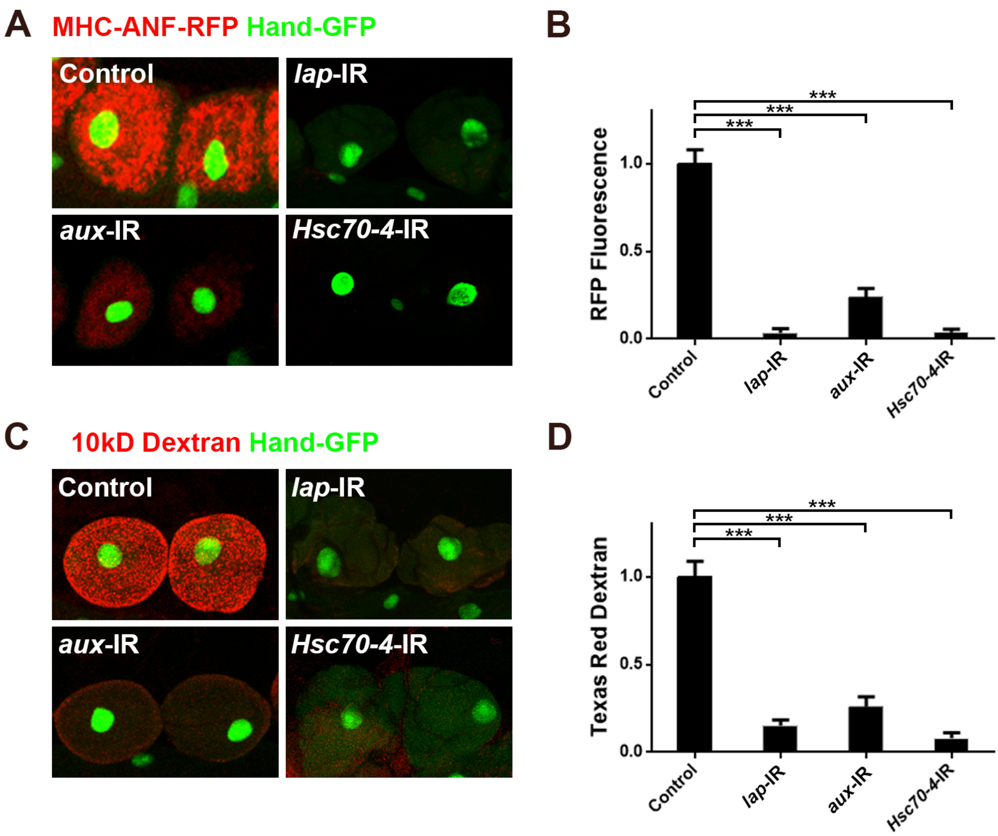

Supplemental Figure 9

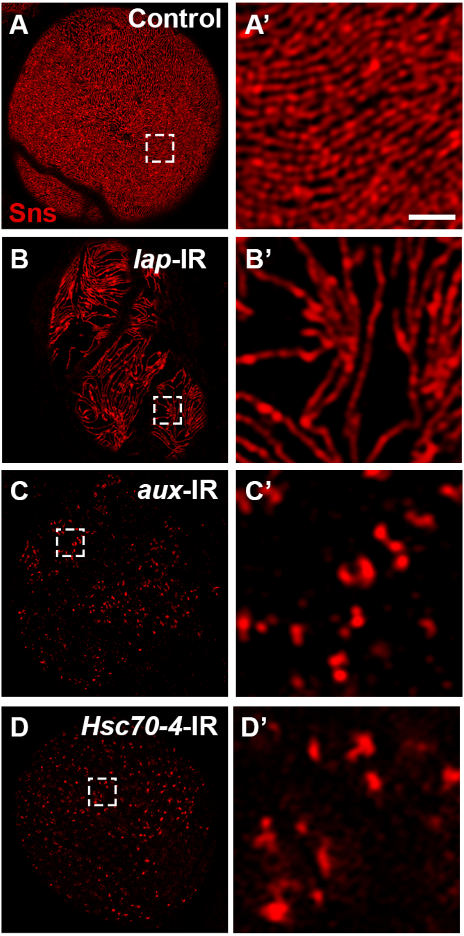

Supplemental Figure 10

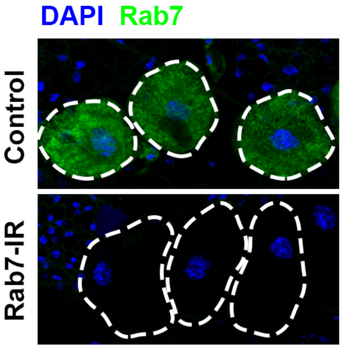

Supplement: Supplementary file 1 — Additional file1: Table S1. The RNAi screen of endocytosis genes required for slit diaphragm protein endocytosis and recycling. Figure S1. The clathrin genes, Chc and Clc, are required for nephrocyte function. A MHC-ANF-RFP derived hemolymph ANF-RFP (red) uptake by nephrocytes. Hand-GFP labels both nephrocytes (big nucleus) and heart cells (small nucleus). Wild type (Control) flies accumulated abundant ANF-RFP. Subsequent panels show that Chc and Clc genes RNAi silencing severely reduced the ANF-RFP accumulation level. B Texas Red-labeled 10 kD Dextran particles uptake by nephrocytes. Particles were easily taken in and collected by wild type control nephrocytes in an ex vivo assay. Silencing of Chc and Clc genes clearly reduced levels of accumulated Dextran. C Quantification of relative ANF-RFP fluorescence in nephrocytes expressing the indicated gene silencing RNAi construct. D Quantification of relative Dextran-linked Texas Red fluorescence in nephrocytes expressing the indicated gene silencing RNAi construct. ***P<0.001. Figure S2. The clathrin genes, Chc and Clc, are required for slit diaphragm protein Sns endocytosis. A-A’ Sns (red) distributed uniformly and smoothly in parallel lines in fingerprint like pattern in control (wild type) nephrocytes. Scale bar: 1μm. B-C’ Silencing of Chc and Clc resulted in severe disruption of Sns cell surface localization. The Sns-positive lines became more curved and shorter, and many became dots. The lines were no longer parallel, and the spacing became irregular. Figure S3. The Drosophila dynamin homolog, Shi, is required for nephrocyte function. A MHC-ANF-RFP derived hemolymph ANF-RFP (red) uptake by nephrocytes. Hand-GFP labels both nephrocytes (big nucleus) and heart cells (small nucleus). Wild type (Control) flies accumulated abundant ANF-RFP. Subsequent panel shows that shi gene RNAi silencing severely reduced the ANF-RFP accumulation level. B Quantification of relative ANF-RFP fluorescence in nephrocytes expressing the [file 13578_2021_595_MOESM1_ESM.pdf]
